# Citizens’ opinions and experiences related to costs and reimbursements for medications in times of retrenchment: cross-sectional population surveys in 2015 and 2017

**DOI:** 10.1186/s12939-022-01631-6

**Published:** 2022-03-09

**Authors:** Katri Aaltonen, Mikko Niemelä, Irene Prix

**Affiliations:** 1 Department of Social Research, University of Turku, Turku, Finland; 2 Kela research, the Social Insurance Institution of Finland, Helsinki, Finland

**Keywords:** Cost of illness, Chronic illness, Financial burden, Legitimacy, Retrenchment, Public opinion

## Abstract

**Background:**

Finland has universal coverage for prescription medications under the National Health Insurance. Eligibility schemes target higher reimbursements to individuals with chronic illness. Nevertheless, co-payments always apply, and austerity reforms implemented in 2016 and 2017 led to further increases in co-payments. We examined the extent to which people with chronic illness experienced financial difficulties in purchasing medications, how perceptions of fairness regarding the national reimbursements differs by exposure to policies and medicine use, and in what way do these experiences and opinions vary between surveys collected before and after the reforms.

**Methods:**

We used two waves of Medicines Barometer (2015 and 2017, pooled *n* = 10,801), a national, biennial, cross-sectional population survey. Logistic regression analyses were performed with experiences of financial difficulties and perceptions of fairness as dependent variables. We compared people with and without prescription medication use, eligibilities, and/or diabetes (exposure groups), controlling for age, gender, survey type and geographic area (NUTS2). To examine the modifying effect of study year, we fitted models with an interaction term between group and year.

**Results:**

Respondents with diabetes or eligibility based on chronic illness had a notably higher risk than other respondents with at least some prescription medication use to have experienced financial difficulties in affording medications. The share of respondents experiencing difficulties increased the most among people with diabetes. Three-quarters of respondents were either critical or unsure of whether the reimbursements for medications were fair and just. People with recent prescription medication use tended to be more sceptical than people without. Overall, scepticism tended to be more prevalent in 2017 than in 2015.

**Conclusions:**

Despite the protective policies in place, individuals with chronic illness were disproportionately burdened by costs of medications already before the reforms. Among individuals with diabetes, financial difficulties were even more prevalent in 2017 than in 2015, which is likely attributed to the particularly high co-payment increases targeted to type 2 diabetes medicines. Perceived fairness of the processes and outcomes of policies and regulations is a key dimension of trust in public policy. Thus, increasing scepticism implies that retrenchment may also have implications in terms of public legitimacy.

**Supplementary Information:**

The online version contains supplementary material available at 10.1186/s12939-022-01631-6.

## Background

In Europe, chronic illnesses are the leading cause of mortality and morbidity. Access to medications is an important part of the care required to address the needs of individuals living with chronic illnesses [1]. Affordable medications are also acknowledged as a key requirement for functional healthcare, to which fair and equitable access is presented as a central pillar of the European lifestyle [2].

Ensuring affordability of medications for patients and health systems is a growing challenge [2, 3]. The ongoing pandemic places sustainability pressures on health systems, many of which are already burdened by more than a decade of fragile economic recovery. At the same time, systems struggle to find ways to adapt to pressures driving cost growth associated with, for example, technological innovation and increasing patient complexity.

While pursuing sustainability without continuing to compromise the guiding values for European health systems, the European Commission’s Expert Panel on effective ways of investing in health (EXPH) stated in their recent *Opinion* that increasing efficiency through the reallocation of resources is of utmost necessity [4]. They defined that value-based healthcare requires tackling inefficiencies (e.g. overdiagnosis and overtreatment) and inequities (e.g. between diseases).

However, the suggested measures require prioritisation and limit setting, which occur in the context of conflicting interests and values, and are therefore a source of controversy among stakeholders [5, 6]. Patients, citizens, and health professionals may disagree on the principles and processes that public payers apply for priority setting, or they may lack trust in the payers to make decisions for the right reasons [7–9]. Debates may increase public dissatisfaction and lower trust in access to care. Perceived security, that is, peace of mind that adequate medical treatment is available when needed, is an important aspect of healthcare responsiveness. Trust and satisfaction are in turn critical in terms of popular legitimacy [10, 11].

In practice, many European countries have responded to fiscal pressures by increasing co-payments; thus, they seem to be one of the most easily implemented policy options [12, 13]. However, co-payments and other cuts in health coverage have inequitable effects. Particularly, poorer individuals in Europe face financial hardship due to costs related to healthcare, and outpatient medications are generally the main drivers behind these problems [14].

In Finland, an increase in medication co-payments has been a recurrent policy used to create savings. Recent studies applying quasi-experimental analytical strategies have shown coinciding negative effects: decreases in the purchasing of medications among patients with schizophrenia and stagnation in the preceding decreasing trend in psychiatric hospitalisations [15]; decreases in the consumption of type 2 diabetes medications and worsening glycaemic control [16], and increases in the use of last-resort social assistance vouchers to pay for type 2 diabetes medications [17]. In longitudinal studies using survey data, increases in financial problems in buying medications and dissatisfaction among people with type 2 diabetes have been observed [18, 19].

We built on previous findings; however, we broadened the respondent base from specific patient groups to a sample representative of the population. We assessed the performance and legitimacy of the national pharmaceutical reimbursement system through citizens’ eyes by surveying their experiences of financial difficulties in buying medications as well as opinions on the fairness of the national reimbursement system. Our study enables contextualising the previous findings to a wider spectrum of chronically ill individuals and to the general public.

Reimbursement policies offer an interesting case for studying public opinions. Healthcare is one of the largest and most valued components of welfare states [11, 20], and retrenchment is expected to be highly unpopular. Nevertheless, retrenchment seems to create policy feedback only if it is very large, recent, and transparent enough [21–24]. Nevertheless, general policy feedback outcomes, such as electoral behaviour, might not be sensitive enough to capture citizens’ reactions toward specific retrenchment policies [25]. In the era of *constant austerity* [26], it may also be less clear to citizens whom to hold accountable [27].

A more direct way of addressing citizens’ opinions is performance evaluations, that is, citizens’ subjective assessments and experiences of benefits and services [22, 28–31]. Negative views and experiences may lower political trust and low trust may influence how citizens view welfare state performance. Consequently, struggles in being able to finance the expected level of benefits and services to citizens can lead to a reciprocal downward spiral of inadequate performance fueling distrust, leading to pessimistic expectation [32].

As health systems prepare to make increasingly difficult decisions regarding prioritisation, political trust is an important resource. Individuals with higher political trust tend to evaluate government decisions, even those that are disadvantageous to them, more positively than individuals with low trust [33]. Monitoring changes in opinions during retrenchment can provide important signals of changes in the public mood [34].

We compared results from population surveys before and after the implementation of two sets of retrenchment policies that increased medication co-payments. Our data and analytical setup did not allow for a more rigorous testing of whether observed changes can be causally attributed to these reforms. However, the policies were relatively transparent, which means the public was aware of the co-payment increases, as they were abrupt and recent, they directly affected a large part of the population, and they were put on the agenda by the stakeholders and opposition parties, leading to widespread criticism in the media.

### Study objectives

We studied citizens’ experiences of financial difficulties purchasing medications and their opinions regarding the fairness of reimbursements in Finland using cross-sectional population survey data collected in 2015 and 2017.

In terms of experiences, the aim was to complement previous findings by using subjective measures, which is typical in the assessment of financial protection [14]. In terms of opinions, we assessed general perceptions of the fairness and justness of reimbursements. Perceived fairness of the processes and outcomes of policies and regulations is a key dimension of trust in public policy [35].

The focus of this study is to compare the experiences and opinions of groups differentially exposed to reimbursements and medication use. First, we examined individuals with *eligibility*, that is, individuals diagnosed with specific chronic illnesses, that are eligible for co-payment reductions in the Finnish system (see Settings of the study for description). However, we further distinguished respondents with diabetes (whom in most cases are also eligible) as a separate group because diabetes medications were targeted by co-payment increases twice. Second, we separated the remaining respondents into two groups based on whether they had used any prescription medications during the previous year. Thus, we examined four groups: people with diabetes, individuals eligible due to chronic illnesses (excl. diabetes), others who used prescription medications, and others who did not use prescription medications.

We were interested in the variation between surveys conducted in 2015 and 2017 because of the co-payment increases implemented during that time. However, it should be noted that our data is cross-sectional (i.e. we did not follow the same individuals over time), and does not allow empirical testing of intra-individual changes over time. Therefore, causal interpretations of the effects of policy changes remain speculative and should be confirmed by studies using a longitudinal design. Overall, the literature on policy feedback has traditionally relied on cross-sectional data because other data are often non-existent [36], which is the case for the phenomenon under examination in this article.

More specific research questions are listed below (See Additional file 1 for conceptual framework):

1. To what extent were people protected from financial difficulties in buying medications through reimbursement policies?
2. How did perceptions of fairness regarding the Finnish reimbursement system differ by groups differentially exposed to reimbursement policies and prescription medication use, that is, people with and without prescription medication use, eligibilities, and/or diabetes?
3. In what way did this exposure to financial difficulties due to prescription medications and the perception of fairness of the reimbursements vary between surveys conducted in 2015 and 2017?

## Methods

### Settings of the study

All residents are universally entitled to social and healthcare services, including prescription medications [37, 38]. Approximately 70% of the population purchases medications that are reimbursable by the National Health Insurance (NHI) annually [39]. Co-payments always apply, and they are not sensitive to income [40]. Individuals diagnosed with specific chronic illnesses are eligible for co-payment reductions under Special Reimbursement schemes. However, access to eligibility requires that the person receives an entitling diagnosis, and enters the application process. Eligibilities are disease-based, which means they only apply to medications used to treat specific diseases (one patient may, however, have several eligibilities) [40, 41]. The medications also need to be on the national positive list, which requires application process initiated by the marketing authorisation holder.

In 2015, the standard co-payment was 65% of the retail price [42]. Reduced co-payments for eligible patients were, depending on their illnesses, either 35% of the retail price (e.g. hypertension, asthma) or €3 fixed fee per item (e.g. diabetes, cancer). The annual co-payment ceiling was €613, after which a fixed fee of €1.50 per item was applied.

In 2016, an annual deductible (€50/year) was implemented [42–45]. This means that patients needed to pay the full cost of medications out-of-pocket up to €50, after which the aforementioned reimbursements were applied for the rest of the calendar year. To compensate for this change, the standard co-payment was reduced from 65 to 60%. The fixed fee increased from €3 to €4.50, and the fee after the annual ceiling increased from €1.50 to €2.50. The reforms increased the average annual co-payment expenditure for reimbursed pharmaceuticals by €12 among users [42]. For 10%, the increase was over €30, and for 3%, the increase was over €50. Larger increases concentrated particularly on individuals with eligibilities.

In 2017, type 2 diabetes medications changed their Special Reimbursement category [16–18, 46, 47]. Prior to the reform, the co-payment for all diabetes medicines included in the Special Reimbursement was a €4.50 fixed fee; and after the reform, it was 35% of the retail price. The reform thus made the co-payment dependent on the price of the product, and accordingly, the anticipated increases were notably larger for patients who used newer, higher price products (estimated mean co-payment increase €157 per year) than for patients who used older, lower price products (estimated mean co-payment increase €12 per year) [48].

Because of the widespread use of newer products (most commonly dipeptidyl peptidase (DPP-)4 inhibitors in combination with metformin), the reform meant notable co-payment increases for many patients, most of whom were older and at the lower end of the income distribution [16–18, 48]. In 2016, 31% of all patients receiving special reimbursement for type 2 diabetes medicines bought DPP-4-inhibitor products, and 13% bought fixed-dose combination products (typically DPP-4-inhibitor with metformin) [39]. The use of the newest products was less prevalent (8% bought sodium-glucose cotransporter (SGLT)2-inhibitors and 5% glucagon-like peptide (GLP)-1-analogues); however, the use of the oldest agents (excluding metformin) was even more rare: 4% bought sulfonylureas, 2% bought thiazolidinediones and 1% bought other agents (incl. meglitinides).

### Data collection

We used pooled data from two waves (2015 and 2017) of the Finnish Medicines Barometer, a national, biennial, cross-sectional population survey examining experiences, opinions, and values related to health, medicines, and well-being [49, 50]. Data were collected by a market research company (Taloustutkimus Ltd) using two methods: a postal survey and an internet panel survey.

The postal surveys were mailed in 2015/2017, respectively, to a random sample of 8,003/8,000 individuals aged 18–80/18 − 79 years from the national population register, stratified at individual level by gender, age, and residential area. After two reminders, the final response rates were 39.9%/45.3%, respectively (*n* = 3,190/3,622).

Internet panel survey respondents were derived from a pre-recruited internet panel containing approximately 40,000 Finns. To achieve the target number of 2,000 respondents, in 2015/2017, respectively, 13,900/8,987 respondents, representing 18 − 79-year-old Finns, stratified by gender, age, and area of residence, were invited to participate, yielding 2,235/2,149 responses. In relation to invitations, the response rates of internet panel surveys were 16.1% and 23.9%, respectively; however, their response rates were not comparable to those of the postal surveys, because of methodological differences.

### Measures of experiences and opinions (outcomes)

Experiences were measured by the following question: “During the last year, have you had financial difficulties in buying medicines prescribed to you by a doctor? i) I haven’t used prescription medicines. ii) I have had no difficulties. iii) I have had some difficulties. iv) I have had plenty of difficulties.” For statistical analyses, we dichotomised options into (ii) *No difficulties* and (iii & iv) *At least some difficulties*.

Opinions on reimbursements were measured by the question: “How do you feel about Kela [= the Social Insurance Institution of Finland] reimbursements for medicine expenses? Please give your opinion on each statement even if you don’t currently use prescription medicines. Reimbursements for medicine expenses are fair and just. i) Fully agree, ii) Fairly agree, iii) Fairly disagree, iv) Fully disagree, v) I don’t know.” For the analyses, we combined options as (i & ii) *Agree*, and (iii & iv) *Disagree*. The share of respondents choosing “I don’t know” was relatively large at over 20%, and it was included as a third category.

### Exposure groups

We defined groups based on differential exposure to reimbursement policies and medication use. Individuals with eligibility have a high need for medications by definition, and they are entitled to higher reimbursements. However, they were also exposed to larger co-payment increases. Individuals without eligibilities may have lower need for medications, and they could be less exposed to prescription costs altogether. However, they may also have illnesses that do not make them eligible and pay a higher share or pay for all of their medications out-of-pocket.

For the analyses, we separated four mutually exclusive groups: (I) respondents with diabetes, (II) respondents with eligibility excl. diabetes, (III) other respondents with at least some prescription medication use during the preceding year, and (IV) other respondents with no prescription medication use during the preceding year.

Individuals with diabetes (group I) were classified based on multiple questions: 95% were classified based on a question related to longstanding illnesses and 5% were classified based on other variables (eligibility code or using diabetes medications during the preceding 2 weeks). Of note, co-payment increases implemented in 2017 only affected individuals who used type 2 diabetes medications, however, we could not reliably distinguish between people using different diabetes medications.

The remaining individuals with eligibility (group II) were classified based on answering “Yes” to the following question: “Do you have an illness that entitles you to the special reimbursement for medicine expenses from Kela?”. Other respondents who answered “No” were further classified as using (group III) or not using (group IV) prescription medications during the preceding year based on the question described in the previous chapter measuring financial difficulties.

### Other independent variables and covariates

We used the study year as a modifier to examine differences between samples collected in 2015 and 2017. As covariates, we used age, gender, geographic area of residence (Nomenclature of Territorial Units, NUTS level 2), and survey collection method (postal/internet panel). These were used to adjust for differences due to attrition across the exposure groups and data collection years. Survey collection method is included, because postal and web surveys can produce slightly different results [51, 52].

Of note, we refrained from controlling for proxies of socioeconomic position to minimise risk of overcontrol, which means removing part of the effect of chronic illness on outcomes [53, 54]. The socioeconomic gradient of health is one of the main mechanisms through which ill health increases the risk of financial burden; thus, an important part of the effect we aim to measure [55]. However, to examine heterogeneity across study years, we conducted sensitivity analyses where we controlled for household income and marital status (to account for household size).

### Statistical methods

First, we examined the effect of exposure to medicine use and reimbursement policies on the probability of experiencing financial difficulties in buying medications using binary logistic regression. The exposure groups were diabetes, eligibility, and other prescription medication use (reference group).

Second, we examined the effect of exposure on the opinions on reimbursements using multinomial logistic regression. The exposure groups were diabetes, eligibility, other prescription medication use and no prescription medication use (reference group).

In all models, we controlled for study year and covariates (gender, categorised age, NUTS2, and survey type). To examine the modifying effect of study year, we fitted models, where we included an interaction term between comparison group and year.

The results of the logit models are presented as average marginal effects (AME) on the predicted outcome probabilities. In terms of effect sizes, AMEs can be interpreted as percentage point (pp) differences in probabilities in relation to the reference group. Unlike logit coefficients and odds ratios, AMEs allow elaboration and interpretation of effect sizes across groups and models [56]. Interaction effects are interpreted by using AMEs and test for statistical significance using the Wald-test (test of second differences) [57].

For the examination of opinions, we used the responses of all participants for whom we had non-missing data on all variables. For the analyses of financial difficulties, we excluded individuals who had not used prescription medications.

In sensitivity analyses, we examined the associations with and without controlling for household income and marital status. The results were in keeping with the main analyses [Additional file 2].

Statistical analyses were performed with STATA 16.1 statistical software (Stata Corporation, College Station, TX, USA), using packages *estout* [58], *spost13_ado* [59], and *mplotoffset* [60].

## Results

### Characteristics of the study population

The study population included 10,801 individuals [Additional file 3]. For the analyses of financial difficulties, we excluded 14% (*n* = 1,553) who had not used prescription medications, thus resulting in a total of 9,268 responses. Individuals with diabetes or eligibility were on average older, had lower level of income, worse health status and a higher prevalence of disabling and/or chronic illnesses than respondents without [Additional file 4]. They also reported higher spending on prescription medicines [Additional file 5].

### Experiences of financial difficulties in buying prescription medications

Table 1 presents the main effects of independent variables and covariates on the probability of having experienced at least some financial difficulties. Respondents with diabetes had 23 percentage points (pp) higher probability than other prescription medication users to have experienced financial difficulties, net of controls. Respondents with eligibility faced a higher risk of financial strain from medicine expenses than respondents with other prescription medicine use, though the difference was on average lower (13 pp) than observed among individuals with diabetes.

**Table 1:** Financial difficulties: marginal effects of independent variables

	AME	se
**Group (ref: Other Rx users)**		
Diabetes	0.231^***^	0.015
Eligibility	0.128^***^	0.010
**Survey year (ref:2015)**		
2017	0.019^*^	0.008
**Gender (ref: Female)**		
Male	-0.048^***^	0.008
**Age (ref: 40–59 years)**		
18–39	0.027^*^	0.013
60–69	-0.027^**^	0.010
70+	-0.064^***^	0.010
**NUTS2 (ref: Western)**		
Helsinki-Uusimaa	-0.007	0.010
Southern	0.018	0.011
Northern/Eastern	0.006	0.011
**Survey type (ref:postal)**		
Internet panel	0.030^***^	0.008
n	9268	
Pseudo R-square	0.053	
AIC	7925	
BIC	8004	

Average marginal effects (AME), with standard error (se) of the independent variables and covariates on the probability of having experienced financial difficulties in buying prescription medications (Rx) during preceding year. Results are based on binary logistic regression (main effects). * *p* < 0.05, ** *p *< 0.01, *** *p *< 0.001

**Fig. 1:**
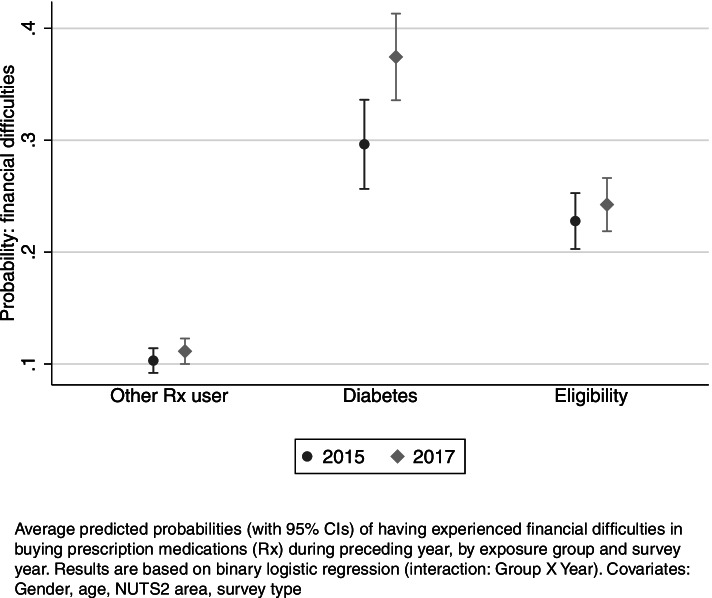
Probability of having experienced financial difficulties in buying medications during preceding year

To examine whether experiences developed differently across years in the examined groups, we tested for the interaction between comparison group and study year in our model. Figure 1 presents the average predicted probabilities of respondents in the comparison groups to have experienced financial difficulties across study years. In both years, respondents with diabetes or eligibility were more likely to experience difficulties than respondents in the reference group. However, the share of respondents experiencing difficulties increased more strongly among people with diabetes compared to respondents in other groups between 2015 and 2017.

Next, we tested whether this change over time in group-specific difficulties to afford medications was statistically significant (Table 2).

**Table 2:** Financial difficulties: differences in the effects of group across study years

	AME	se
**Group (ref: Other Rx user)**		
a Diabetes, 2015	0.197^***^	0.022
b Diabetes, 2017	0.259^***^	0.020
c Eligibility, 2015	0.128^***^	0.014
d Eligibility, 2017	0.128^***^	0.013
**Contrasts**		
b-a Diabetes 2017 vs. 2015	0.061^*^	0.029
d-c Eligibility 2017 vs. 2015	0.001	0.019
n	9268	
Pseudo R-squared	0.054	
AIC	7908	
BIC	7936	

Average marginal effects (AMEs), with standard error (se), of exposure group on the probability of having experienced financial difficulties in buying prescription medications (Rx), and differences in the effects of group across study years. *Contrasts* report statistical tests for differences over time in the marginal effects of exposure group vs. reference group. Results are based on binary logistic regression (interaction: Group X Year). Covariates: Gender, age, NUTS2, survey type. ^*^ *p* < 0.05, ^**^ *p* < 0.01, ^***^ *p* < 0.001

The risk of people with diabetes experiencing financial difficulties increased statistically significantly by 6 pp between 2015 and 2017: in 2015, people with diabetes were 20 pp more likely than other prescription medication users to face difficulties, but by 2017 this difference in risk had risen to 26 pp. In turn, eligible respondents had 13 pp higher probability of having experienced difficulties than respondents in the reference group in both years; thus, the effect of eligibility was not significantly different across years.

In sum, respondents with diabetes or eligibility had a notably higher risk than other respondents with at least some prescription medication use to experience financial difficulties in both years, and the share of respondents experiencing difficulties in affording medications increased the most among people with diabetes.

### Opinions on the fairness of reimbursement policies

Table 3 presents the results regarding the probability of agreeing, disagreeing or being unsure about the fairness of the reimbursements.

**Table 3:** Perceived fairness: marginal effects of independent variables

	Agree		Disagree		Don’t know	
	**AME**	**se**	**AME**	**se**	**AME**	**se**
**Group (ref: No Rx use)**						
Others with Rx use	0.026^*^	0.013	0.094^***^	0.014	-0.120^***^	0.014
Diabetes	0.027	0.018	0.186^***^	0.019	-0.213^***^	0.017
Eligibility	0.054^***^	0.016	0.117^***^	0.016	-0.171^***^	0.015
**Survey year (ref: 2015)**						
2017	-0.083^***^	0.009	0.063^***^	0.009	0.019^*^	0.008
**Gender (ref: Female)**						
Male	0.081^***^	0.009	-0.044^***^	0.009	-0.037^***^	0.008
**Age (ref: 40–59 years)**						
18–39	0.125^***^	0.013	-0.156^***^	0.013	0.031^**^	0.011
60–69	0.003	0.012	0.019	0.013	-0.022^*^	0.010
70+	0.038^**^	0.013	-0.082^***^	0.014	0.044^***^	0.012
**NUTS2 (ref: Western)**						
Helsinki-Uusimaa	< 0.001	0.012	-0.029^*^	0.013	0.029^*^	0.011
Southern	-0.006	0.013	0.004	0.013	0.003	0.011
Northern/Eastern	0.022	0.013	-0.002	0.013	-0.020	0.011
**Survey type (ref: Postal survey)**						
Internet panel	0.025^**^	0.009	0.039^***^	0.010	-0.063^***^	0.008
n	10,801					
Pseudo R-square	0.03					
AIC	22,474					
BIC	22,561					

Average marginal effects (AMEs), with standard error (se), of the independent variables on the probability of agreeing, disagreeing and being unsure with statement “reimbursements are fair and just”. Results are based on multinomial logistic regression (main effects). Rx = Prescription medications. * *p *< 0.05, ** *p* < 0.01, *** p < 0.001

Compared to respondents with no prescription medication use, individuals in other groups were overall more sceptical about the fairness of the reimbursement system. This scepticism was most pronounced for people with diabetes, who were about 19 pp more likely than respondents without prescription medication use to disagree with the statement that the reimbursement system is fair and just. However, this does not mean that respondents without prescriptions perceived the reimbursement system as fair. Instead, they appeared to feel less able to judge the fairness of the system altogether.

**Fig. 2:**
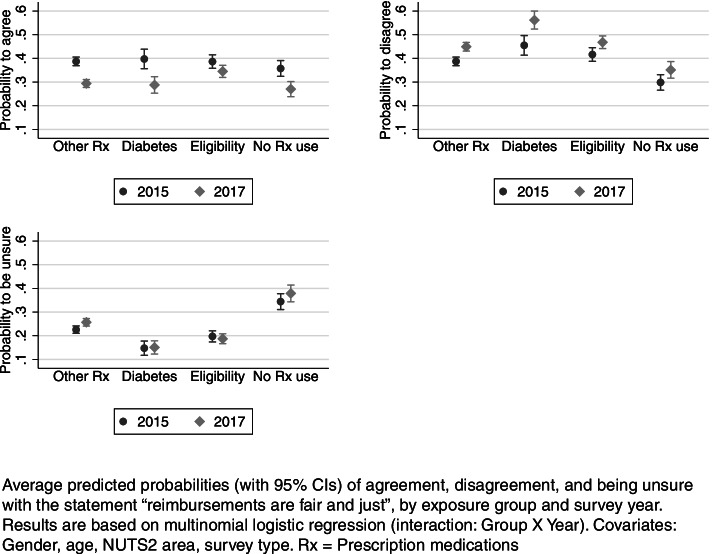
Probability of agreement, disagreement, and being unsure with the statement “reimbursements are fair and just”

Next, we examined whether the perceived fairness may have disproportionately changed over time for groups most affected by the reforms. Figure 2 presents year-specific average predicted probabilities estimated by the interaction model for patient groups in 2015 and 2017. The results show that respondents’ positive evaluations decreased alongside an increase in negative views. These changes appear to have affected different respondent groups in a similar way. A noteworthy exception to this trend concerns respondents with eligibilities. Among them, positive fairness perceptions of reimbursement policies remained fairly stable, whereas they decreased more markedly among respondents with diabetes and other prescription medicines.

To more formally assess whether fairness perceptions of reimbursement policies changed more strongly among patient groups affected by policy reforms, Table 4 presents the year-specific average marginal effects of exposure estimated by our interaction model as well as tests of their difference between 2015 and 2017.

**Table 4:** Perceived fairness: differences in the effects of group across study years

	Agree		Disagree		Don’t know	
	**AME**	**se**	**AME**	**se**	**AME**	**se**
**AME of group (ref: No Rx use, no eligibility, no diabetes)**						
a Other Rx users 2015	0.030	0.019	0.088^***^	0.019	-0.118^***^	0.019
b Other Rx users 2017	0.024	0.018	0.099^***^	0.020	-0.122^***^	0.020
c Diabetes 2015	0.040	0.027	0.156^***^	0.027	-0.196^***^	0.023
d Diabetes 2017	0.017	0.024	0.211^***^	0.027	-0.229^***^	0.023
e Eligibility 2015	0.029	0.023	0.117^***^	0.022	-0.146^***^	0.021
f Eligibility 2017	0.075^***^	0.021	0.118^***^	0.023	-0.192^***^	0.021
**Contrasts**						
b-a: Other Rx 2017 vs. 2015^a^	-0.006	0.026	0.010	0.028	-0.004	0.027
d-c: Diabetes 2017 vs. 2015^a^	-0.023	0.036	0.056	0.037	-0.033	0.032
f-e: Eligibility 2017 vs. 2015^a^	0.045	0.030	< 0.001	0.031	-0.046	0.029
(d-c)-(b-a) Diab. vs. Other Rx^b^	-0.017	0.030	0.045	0.031	-0.029	0.024
(d-c)-(f-e) Diab. vs. Elig.^b^	-0.068^*^	0.033	0.055	0.034	0.013	0.026
(b-a)-(f-e) Other Rx vs. Elig.^b^	-0.051^*^	0.023	0.010	0.024	0.042^*^	0.019
n	10,801					
Pseudo R-square	0.031					
AIC	22,465					
BIC	22,552					

Average marginal effects (AMEs), with standard error (se), of exposure group on the probability of agreeing, disagreeing and being unsure with the statement ‘reimbursements are fair and just’. *Contrasts* report statistical tests for differences over time in the marginal effects of exposure group vs. reference group(^a^), and the other groups vs. each other(^b^). Results are based on multinomial logistic regression (interaction: Group X Year). Covariates: Gender, age, NUTS2, survey type. Rx = prescription medicines. ^*^ *p* < 0.05, ^**^ *p* < 0.01, ^***^ *p* < 0.001

In both years, chronic disease and eligibility status made little (and mostly statistically nonsignificant) difference on the probability to agree with the statement. However, in 2017, people with eligibility had 7.5 pp higher probability to agree than people in the reference group (*p* < 0.001). The probability to disagree was significantly higher, and the probability to be unsure significantly lower, among respondents with diabetes, eligibility, or other prescription medication use in comparison to the reference group in both years (Table 4, upper panel).

However, we mostly find no statistically significant changes over time in this relationship (Table 4, lower panel). In other words, while perceptions of the reimbursement policies’ fairness took a more negative turn overall, differences in opinions, in relation to the reference group, remained fairly similar. However, if we compare changes over time in relation to respondents with eligibility (rather than individuals with no medication use), we find a slightly more pronounced waning of diabetes patients’ (-7 pp) and other prescription users’ (-5 pp) fairness perceptions (Table 4, lower panel). This is due to the opinions of respondents with eligibility being slightly more stable over time than the opinions of respondents with diabetes or other prescription medicine use (see also Fig. 2).

In sum, respondents with recent experience of prescription medication use tended to be more sceptical about the fairness of the reimbursement system than other respondents, which in turn tended to be more often unsure of their opinions. The perceptions of fairness were more negative in 2017 than in 2015, however, the differences in opinions between exposure groups remained relatively similar.

## Discussion

We examined the performance of the Finnish reimbursement system based on the experiences and opinions of citizens. Approximately one-sixth of those who used prescription medications experienced financial difficulties purchasing them, and these difficulties were notably more prevalent among individuals with eligibility or diabetes than among other respondents with prescription medication use. In terms of opinions, a larger part of the respondents disagreed with the statement that reimbursements are fair and just; however, a considerable part was also unsure of their opinion. People with recent prescription medication use tended to be more sceptical than people without, and scepticism tended to be more prevalent in 2017 than in 2015.

Eligibility schemes target individuals with chronic illnesses who are deemed at the highest need for pharmacotherapy in the long term. However, approximately every fifth to sixth respondent with recent prescription medication use but no eligibility also reported having moderate-to-poor health status, limiting illness, or high spending on prescription medications [Additional files 4–5]. Nevertheless, those with eligibility and/or diabetes were even more likely to experience financial difficulties purchasing medications than other prescription medication users. Thus, in protecting individuals with chronic illnesses from being disproportionately affected by the financial burden of medications, eligibility schemes do not seem comprehensive and/or generous enough.

Between the data collection periods in 2015 and 2017, implemented austerity policies increased co-payments. We found that the probability of experiencing financial problems was significantly higher among respondents with diabetes in the 2017 survey than in the 2015 survey. However, for the other groups with prescription medication use, the probability did not significantly vary across years.

Several reasons could explain why co-payment increases were reflected in the increasing prevalence of financial difficulties only among respondents with diabetes: (1) The 2017 reform led to larger co-payment increases. (2) They may have been particularly vulnerable because of their high comorbidity and low income. (3) They had experienced increases more recently at the time of the second survey.

It should also be noted that the group of respondents with eligibility is more heterogeneous than the diabetes group; thus, focusing on another high-risk patient group might have revealed further differences across years. For example, patients with severe mental health disorders also seem at high risk based on previous evidence [15, 42]. However, this patient group was small and was largely undetectable in our survey. Thus, other methods are needed to identify smaller patient groups at risk, particularly those who might not have strong political representation to make their voice heard.

Given the well-known risks related to co-payment increases, recognized even in the Governments’ own proposals [43, 47], it may seem difficult to understand why the reforms were implemented in the first place. Moreover, the inequitable effects of high co-payments have been a concern in Finland for long [38, 61]. However, many of the suggested measures promoting rational use of medicines [62–65] have been waiting for a comprehensive reform of the healthcare system, which has been in the making for decades, due to institutional inertia and lack of political compromise. In the meantime, governments seeking savings during their four-year terms have resorted to quicker solutions.

The Finnish case also highlights the trade-off between equity and access to innovation. The patients have had relatively fast access to novel pharmacotherapies, however, leading to a rapid growth in public pharmaceutical expenditure, which was one of the justifications for increasing co-payments [47, 66, 67]. After the reform, patient’s ability to pay is likely to play a larger role in making therapeutic choices. Socioeconomic differences in use patterns have already been observed in other therapeutic areas [68, 69]. Growing differences can further strengthen the “parallel-funding” -driven development towards a *de facto* tiered health system, which goes against the general egalitarian health policy goals [37, 70, 71].

In relation to opinions, the increasing scepticism among respondents with diabetes was anticipated based on previous results [18, 19]. However, we were also able to compare their opinions to those of other respondents, particularly eligible respondents, who share many characteristics and also have a high need for prescription medications.

In 2015, the opinions of respondents with diabetes and eligibility were largely aligned, whereas a more noticeable gap between these groups emerged in 2017. This may partly be due to the increasing prevalence of financial difficulties among individuals with diabetes. However, disagreement on the fairness of the reimbursements was much more widespread than financial difficulties; thus, they are likely to reflect dissatisfaction in a wider sense. People with diabetes might have perceived savings measures targeted to them only as particularly unfair, whereas individuals with other eligibilities may have been relieved for not being targeted by cuts themselves in 2017. Data collection in late 2017 might have also been too late to capture any short-term dissatisfaction among the eligible after the cuts implemented during early 2016. Whether the results observed in this study reflect short-term fluctuation, or longer-term development of opinions, is beyond the scope of this study; however, it is important to address this in further research.

Our results also suggest that dissatisfaction became more prevalent among those who were not personally affected. This provides some support for the common hypothesis of public dissatisfaction toward retrenchment in healthcare. Previous research on electoral punishment showed that reactions were stronger among those directly affected but not limited to them alone [23].

Studies on the dynamics of political trust and citizens’ opinions also suggest that opinions have become more volatile in the short term, reflecting more active policy feedback of increasingly educated and better-informed citizens as well as more critical media coverage [72]. Collective evaluations through media and elites [22] are likely to be particularly influential among those who have personal experience and knowledge of the system. However, it should be noted that at least when the public has a relatively low knowledge level of the system, perceived performance may be prone to change as a result of exposure to whatever information they come across [73]. The available information may also be systematically skewed because mass media often displays a negative bias.

Opinions toward the fairness of the system were nevertheless relatively sceptical in 2015 before the reforms. In light of such harsh evaluations, it is worth considering what exactly does fair and just mean for citizens in this context.

In the survey, the question was imbedded in a set of statements related to co-payments. Therefore, they may have directed respondents’ thoughts. In relation to the egalitarian goals of Finnish welfare policies, several studies and reports using survey and register data have highlighted inequities in access to care and a skewed distribution of direct healthcare payment expenditures concentrating on vulnerable population groups [37, 38, 74].

The formulation of the question also included the name of the institution administering reimbursements (*Kela*), which generally tends to be evaluated more negatively by individuals without than with recent personal experience [75]. *Kela* might also have reminded people about examples of individual patients denied public funding of novel expensive therapies presented in the media, in which public payers are typically framed as hurdles between patients and innovations [76]. This type of media coverage is common in Finland and worldwide and can be a powerful driver of public opinion [77–79]. Decisions influenced through media can nevertheless increase inequities between diseases because some diseases and patient groups are viewed more easily as deserving and have strong advocacy groups, while others remain voiceless [4].

Negative perceptions of fairness may also be attributed to processes that are viewed as key to legitimacy [4, 7]. For instance, publicity and transparency are presented as key conditions for legitimate decision making. However, the Finnish process is relatively opaque because the reimbursement dossiers submitted by pharmaceutical companies and the Ministry’s justifications for reimbursement decisions are confidential.

Public opinion is important in regards to acceptability, legitimacy, and satisfaction with public institutions [80, 81]. In Finland, the Ministry of Social Affairs and Health [82] defines that “the objective of developing pharmaceutical services and the legislation that applies to pharmacotherapy is to guarantee effective, safe, high quality, equitable and cost-effective pharmacotherapy for everyone who needs it.” In light of our results, the objective of equitable access is not entirely achieved, and the perception of the fairness of reimbursements is sceptical. In designing further reforms, policymakers should be aware that retrenchment may have implications in terms of legitimacy and public trust.

Public trust may also play an important role in conditioning the effectiveness of public health measures [83]. Currently, the containment of the COVID-19 pandemic has, in many countries, relied heavily on the populations making a number of behaviour changes voluntarily. Vaccine hesitancy has, for instance, been an important hurdle [84, 85]. It has been linked with belief in conspiracy theories, and trust in authorities seems to mediate the relationship [86–90].

A few caveats regarding this study need to be noted. First, the cross-sectional nature of our data prevents assessments of causality. Therefore, it cannot be confirmed to what extent the differences between years were due to the effects of policies and due to other factors and/or unobserved heterogeneity across samples. We also only had data from two years; therefore, longer trends cannot be observed. Second, several risks of bias exist when self-reported measures are used.

## Conclusions

The reimbursement system does not seem to fully protect individuals with chronical illnesses from being disproportionately burdened financially by the costs of medications. Furthermore, three-quarters of respondents were either critical or unsure of whether the reimbursements for medications were fair and just. Implemented retrenchment policies seemed to coincide with a higher prevalence of scepticism towards the fairness of the system largely across all respondent groups, although the prevalence of financial difficulties seemed to have increased only among individuals with diabetes. In planning future reforms, policymakers should pay particular attention to questions of legitimacy because low trust might make further changes even more prone to public resistance.

## Supplementary Information

**Additional file 1. **Contextual framework.

**Additional file 2. **Sensitivity analyses.

**Additional file 3. **Flow diagram of study participants.

**Additional file 4. **Health status, survey type, and socio-demographic characteristics of the study population in total and by exposure group in the pooled 2015 and 2017 data.

**Additional file 5. **Use of and spending on prescription medicines, and opinions on the fairness of the reimbursement system in the study population in total and by exposure group in the pooled 2015 and 2017 data.

## Data Availability

The data that support the findings of this study are available from the Finnish Medicines Agency Fimea, but restrictions apply to the availability of these data, which were used under permission (FIMEA/2020/005050) for the current study. The authors have no permission to share the data, but access can be sought for research purposes from Fimea (www.fimea.fi).
