# Supplementary figures and images for: Citizens’ opinions and experiences related to costs and reimbursements for medications in times of retrenchment: cross-sectional population surveys in 2015 and 2017
Source: Int J Equity Health. 2022 Mar 9;21:33. doi: 10.1186/s12939-022-01631-6 (PMC8905281; doi:10.1186/s12939-022-01631-6)

**Additional file 1.** Conceptual framework for analyses.

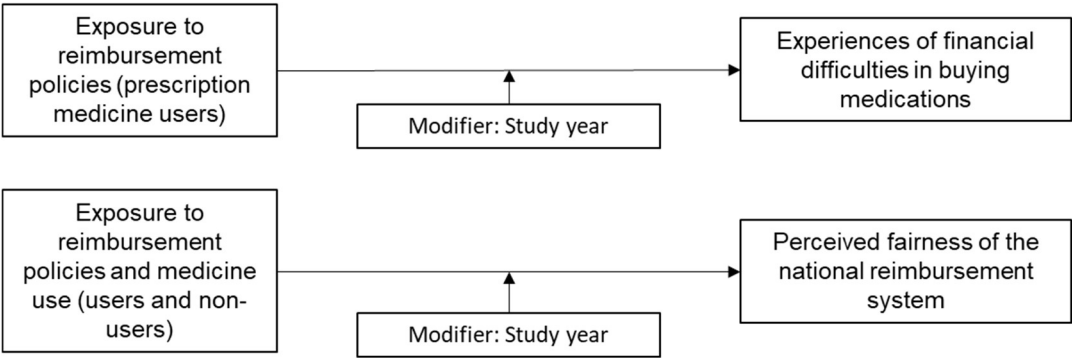

Supplement: Supplementary file 1 — Additional file 1. Contextual framework. [file 12939_2022_1631_MOESM1_ESM.pdf]

**Additional file 3.** Flow diagram of study participants.

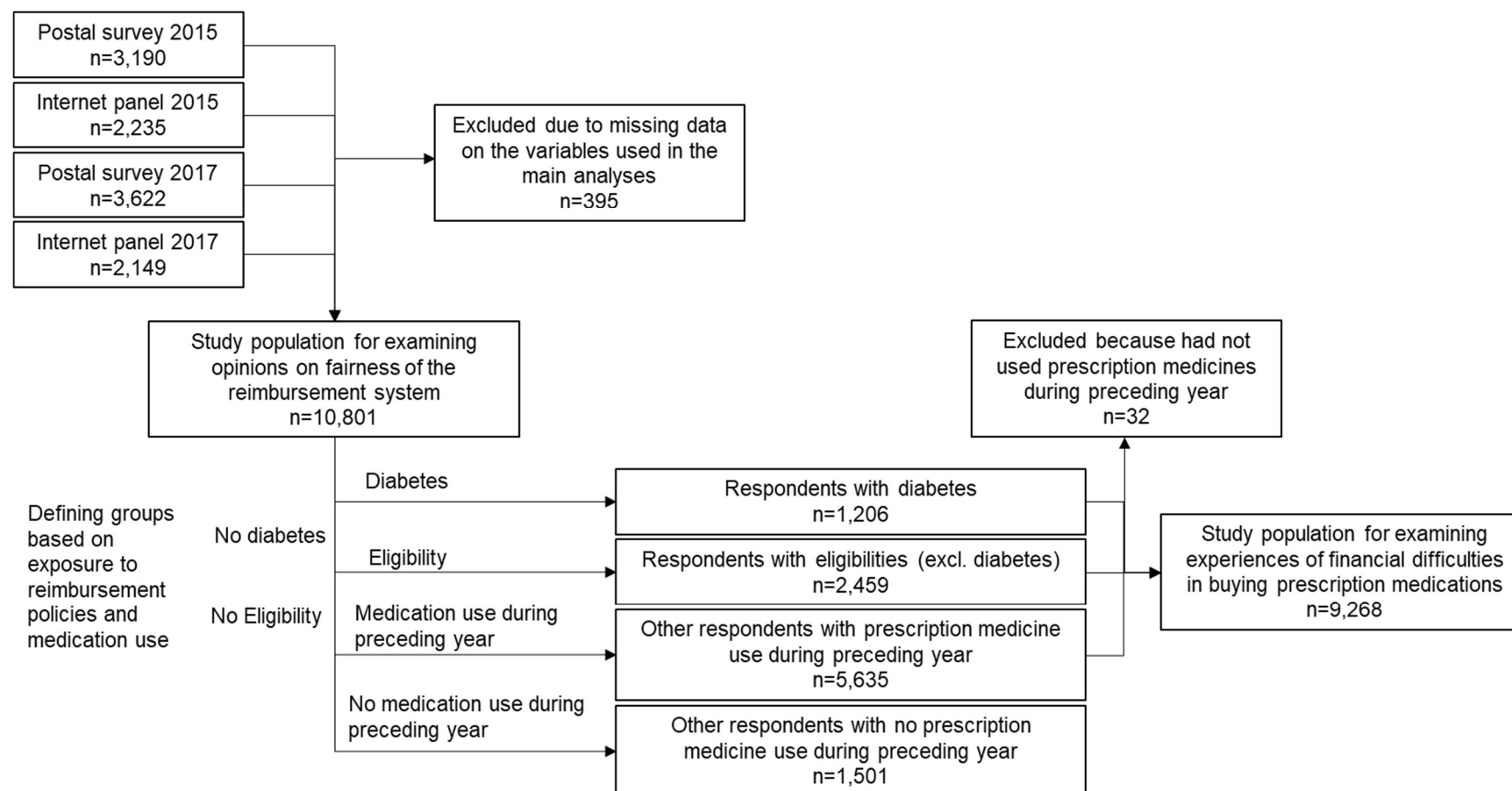

Supplement: Supplementary file 3 — Additional file 3. Flow diagram of study participants. [file 12939_2022_1631_MOESM3_ESM.pdf]
