# Supplementary material for: Citizens’ opinions and experiences related to costs and reimbursements for medications in times of retrenchment: cross-sectional population surveys in 2015 and 2017
Source: Int J Equity Health. 2022 Mar 9;21:33. doi: 10.1186/s12939-022-01631-6 (PMC8905281; doi:10.1186/s12939-022-01631-6)
Supplement: Supplementary file 2 — Additional file 2. Sensitivity analyses. [file 12939_2022_1631_MOESM2_ESM.pdf]

Additional file 2: Sensitivity analyses

In the sensitivity analyses, we examined the association between exposure group and outcomes with (Model B) and without (Model A) controlling for household income and marital status. The study population for sensitivity analyses excluded responses of individuals who had missing data on household income or marital status (*n*=200).

Supplementary tables S1–S4 report the results of the analyses. In terms of experiences of financial difficulties, controlling for income slightly decreased the average marginal effects of diabetes and eligibility (Table S1 and S2). This was in line with our expectation, that controlling for proxies of socioeconomic position removes part of the effect of chronic illness (overcontrol). In terms of opinions, and the modifying effect of study year (test of second differences) in both examinations, controlling for income had only small or negligible effects (Tables S2–S4).

Table S1. Financial difficulties: marginal effects of independent variables.

	**Model A**		**Model B**	
	**AME**	**se**	**AME**	**se**
**Group (ref: Other Rx users)**				
Diabetes	0.232***	0.015	0.193***	0.014
Eligibility	0.127***	0.010	0.115***	0.009
**Survey year (ref:2015)**				
2017	0.020**	0.008	0.028***	0.007
**Gender (ref: Female)**				
Male	-0.049***	0.008	-0.030***	0.007
**Age (ref: 40-59 years)**				
18-39	0.028*	0.013	-0.010	0.012
60-69	-0.026**	0.010	-0.043***	0.010
70+	-0.064***	0.010	-0.092***	0.010
**NUTS2 (ref: Western)**				
Helsinki-Uusimaa	-0.006	0.010	0.018	0.010
Southern	0.017	0.011	0.021*	0.010
Northern/Eastern	0.005	0.011	0.003	0.010
**Survey type (ref:postal)**				
Internet panel	0.031***	0.008	0.043***	0.008
**Household income (ref: €2,000 or less)**				
€2,001-€4,000			-0.165***	0.012

€4,001 or more		-0.257***	0.012
**Marital status (ref:Other)**			
Couple		0.012	0.009
n	9094	9094	
Pseudo R-square	0.053	0.124	
AIC	7779	7209	
BIC	7858	7308	

Average marginal effects (AME), with standard error (se) of the independent variables and covariates on the probability of having experienced financial difficulties in buying prescription medications (Rx) during preceding year. Results are based on binary logistic regression (main effects). * p<0.05, ** p<0.01, *** p<0.001

Table S2: Financial difficulties: differences in the effects of group across study years.

	**Model A**		**Model B**	
	**AME**	**se**	**AME**	**se**
**Group (ref: Other Rx user)**				
a Diabetes, 2015	0.195***	0.022	0.160***	0.020
b Diabetes, 2017	0.263***	0.021	0.220***	0.019
c Eligibility, 2015	0.125***	0.014	0.110***	0.014
d Eligibility, 2017	0.129***	0.013	0.119***	0.013
**Contrasts (second differences)**				
b-a Diabetes 2017 vs 2015	0.068*	0.029	0.060*	0.027
d-c Eligibility 2017 vs 2015	0.004	0.019	0.009	0.018
n	9094		9094	
Pseudo R-squared	0.054		0.124	
AIC	7781		7211	
BIC	7881		7332	

Average marginal effects (AMEs), with standard error (se), of exposure group on the probability of having experienced financial difficulties in buying prescription medications (Rx), and differences in the effects of group across study years. Contrasts report year differences by group (second differences). Results are based on multinomial logistic regression (interaction: Group X Year). Covariates: Gender, age, NUTS2, survey type, and in Model B, marital status and household income. * p<0.05, ** p<0.01, *** p<0.001

Table S3. Perceived fairness: marginal effects of independent variables.

	Agree				Disagree				Don't know			
	Model A		Model B		Model A		Model B		Model A		Model B	
	AME	se	AME	se	AME	se	AME	se	AME	se	AME	se
**Group (ref: No Rx use)**												
Others with Rx use	0.027*	0.013	0.021	0.013	0.094***	0.014	0.096***	0.014	-0.121***	0.014	-0.118***	0.014
Diabetes	0.024	0.019	0.030	0.019	0.190***	0.019	0.184***	0.019	-0.214***	0.017	-0.214***	0.017
Eligibility	0.056***	0.016	0.056***	0.016	0.118***	0.016	0.117***	0.016	-0.173***	0.015	-0.172***	0.015
**Survey year (ref: 2015)**												
2017	-0.083***	0.009	-0.086***	0.009	0.062***	0.009	0.062***	0.009	0.021*	0.008	0.024**	0.008
**Gender (ref: Female)**												
Male	0.083***	0.009	0.073***	0.009	-0.047***	0.010	-0.041***	0.010	-0.036***	0.008	-0.032***	0.008
**Age (ref: 40-59 years)**												
18-39	0.126***	0.013	0.144***	0.013	-0.158***	0.013	-0.169***	0.013	0.032**	0.012	0.024*	0.012
60-69	0.005	0.012	0.018	0.012	0.017	0.013	0.006	0.013	-0.022*	0.010	-0.024*	0.010
70+	0.041**	0.013	0.061**	0.013	-0.084***	0.014	-0.099***	0.014	0.043***	0.012	0.038**	0.012
**NUTS2 (ref: Western)**												
Helsinki-Uusimaa	-0.002	0.013	-0.015	0.013	-0.030*	0.013	-0.019	0.013	0.032**	0.011	0.034**	0.011
Southern	-0.008	0.013	-0.009	0.013	0.003	0.014	0.004	0.013	0.005	0.012	0.005	0.012
Northern/Eastern	0.021	0.013	0.025	0.013	-0.003	0.013	-0.006	0.013	-0.018	0.011	-0.019	0.011
**Survey type (ref: Postal survey)**												
Internet panel	0.024*	0.009	0.017	0.009	0.036***	0.010	0.041***	0.010	-0.060***	0.008	-0.058***	0.008
**Household income (ref: €2,000 or less)**												
€2,001-€4,000			0.042***	0.012			-0.024	0.013			-0.018	0.011
€4,001 or more			0.141***	0.016			-0.110***	0.016			-0.032*	0.014
**Marital status (ref:Other)**												
Couple			-0.008	0.012			0.033**	0.012			-0.025*	0.010
n	10601		10601		10601		10601		10601		10601	
Pseudo R-square	0.031		0.036		0.031		0.036		0.031		0.036	
AIC	22032		21915		22032		21915		22032		21915	

BIC	22120	22024	22120	22024	22120	22024

Average marginal effects (AMEs), with standard error (se), of the independent variables on the probability of agreeing, disagreeing and being unsure with statement "reimbursements are fair and just". Results are based on Multinomial logistic regression (main effects). Rx=Prescription medications. * p<0.05, ** p<0.01, *** p<0.001

Table S4. Perceived fairness: differences in the effects of group across study years.

	**Agree**				**Disagree**				**Don't know**			
	**Model A**		**Model B**		**Model A**		**Model B**		**Model A**		**Model B**	
	**AME**	**se**	**AME**	**se**	**AME**	**se**	**AME**	**se**	**AME**	**se**	**AME**	**se**
**AME of group (ref: No Rx use, no eligibility, no diabetes)**												
a Other Rx users 2015	0.033	0.019	0.025	0.019	0.088***	0.019	0.091***	0.019	-0.120***	0.019	-0.116***	0.019
b Other Rx users 2017	0.021	0.019	0.018	0.019	0.100***	0.020	0.101***	0.020	-0.121***	0.020	-0.119***	0.020
c Diabetes 2015	0.040	0.027	0.045	0.028	0.159***	0.027	0.154***	0.027	-0.199***	0.023	-0.199***	0.023
d Diabetes 2017	0.010	0.024	0.019	0.025	0.218***	0.027	0.210***	0.027	-0.228***	0.024	-0.229***	0.023
e Eligibility 2015	0.030	0.023	0.030	0.023	0.118***	0.022	0.118***	0.022	-0.149***	0.021	-0.148***	0.021
f Eligibility 2017	0.077***	0.021	0.078***	0.021	0.118***	0.023	0.116***	0.023	-0.195***	0.021	-0.194***	0.021
**Contrasts (Second differences)**												
b-a: Other Rx users 2017 vs 2015	-0.011	0.027	-0.007	0.027	0.012	0.028	0.010	0.028	-0.001	0.027	-0.003	0.027
d-c: Diabetes 2017 vs 2015	-0.030	0.036	-0.026	0.036	0.059	0.038	0.056	0.038	-0.029	0.033	-0.030	0.032
f-e: Eligibility 2017 vs 2015	0.047	0.031	0.049	0.031	<-0.001	0.032	-0.002	0.032	-0.046	0.030	-0.046	0.029
n	10601		10601		10601		10601		10601		10601	
Pseudo R-square	0.031		0.036		0.031		0.036		0.031		0.036	
AIC	22022		21905		22022		21905		22022		21905	
BIC	22109		22014		22109		22014		22109		22014	

Average marginal effects (AMEs), with standard error (se), of exposure group on the probability of agreeing, disagreeing and being unsure with the statement 'reimbursements are fair and just'. *Contrasts* report year differences by group (second differences). Results are based on multinomial logistic regression (interaction: Group X Year). Covariates: Gender, age, NUTS2, survey type, and in Model B, marital status and household income. Rx= prescription medicines. * p<0.05, ** p<0.01, *** p<0.001
