# Supplementary material for: Citizens’ opinions and experiences related to costs and reimbursements for medications in times of retrenchment: cross-sectional population surveys in 2015 and 2017
Source: Int J Equity Health. 2022 Mar 9;21:33. doi: 10.1186/s12939-022-01631-6 (PMC8905281; doi:10.1186/s12939-022-01631-6)
Supplement: Supplementary file 4 — Additional file 4. Health status, survey type, and socio-demographic characteristics of the study population in total and by exposure group in the pooled 2015 and 2017 data. [file 12939_2022_1631_MOESM4_ESM.pdf]

Additional file 4. Health status, survey type, and socio-demographic characteristics of the study population (n=10,801) in total and by exposure group in the pooled 2015 and 2017 data.

		Exposure to reimbursement policies and medicine use (exposure groups)			
	**All**	**Diabetes**	**Eligibility (excl. diabetes)**	**Others, with prescription medicine use[a]**	**Others, no prescription medicine use[a]**
Total, row % (n)	100% (10,801)	11% (1,206)	23% (2,459)	52% (5,635)	14% (1,501)
Self-rated health, column % of non-missing (n)					
Good	36% (3,851)	11 %	20 %	40 %	64 %
Pretty good	39% (4,192)	38 %	40 %	41 %	29 %
Moderate	21% (2,259)	40 %	33 %	16 %	6 %
Pretty poor or poor	4% (465)	11 %	7 %	3 %	<1%
All non-missing, n	*10,767*				
missing, n	*34*				
Limiting illness, column % of non-missing (n)					
No	73% (7,805)	52 %	53 %	81 %	94 %
Yes	27% (2,850)	48 %	47 %	19 %	5 %
All non-missing, n	*10,655*				
missing, n	*146*				
Diagnosed long-term illness[b], column % of non-missing (n)					
No long-term illness	35% (3,709)	<1%	2 %	44 %	85 %
Asthma	10% (1,015)	14 %	25 %	4 %	1 %
Hypertension	29% (3,087)	68 %	46 %	20 %	1 %
High cholesterol	17% (1,854)	45 %	24 %	13 %	2 %
Heart disease	9% (947)	21 %	19 %	4 %	1 %
Cancer	4% (373)	6 %	9 %	2 %	1 %
Arthritis or other musculosceletal	16% (1,694)	24 %	25 %	14 %	2 %
Depression or other mental health	8% (816)	9 %	8 %	8 %	4 %
Skin disease	6% (602)	7 %	7 %	6 %	1 %
Gastrointestinal disease	5% (510)	7 %	9 %	3 %	1 %
Other long-term illness	14% (1,545)	12 %	27 %	12 %	3 %
All non-missing, n	*10,661*				
Missing, n	*140*				
Gender, column % (n)[c]					
Female	54% (5,880)	43 %	57 %	59 %	44 %
Male	46% (4,921)	57 %	43 %	41 %	56 %

Age, column % (n)[c]					
18-39	20% (2,154)	2%	8%	25%	35%
40-59	33% (3,560)	23%	28%	37%	34%
60-69	27% (2,923)	40%	32%	24%	21%
70 or older	20% (2,164)	34%	31%	15%	10%
Geographic area of residence (NUTS2), column % (n)[c]					
Western Finland	26% (2,775)	27%	26%	26%	26%
Helsinki-Uusimaa	27% (2,896)	25%	25%	28%	26%
Southern Finland	23% (2,497)	22%	22%	24%	22%
Northern/Eastern Finland	24% (2,633)	26%	27%	22%	26%
Survey year, column % (n)[c]					
2015	48% (5,221)	45%	45%	49%	52%
2017	52% (5,580)	55%	55%	51%	48%
Survey type, column % (n)[c]					
Postal	60% (6,428)	61%	61%	58%	61%
Internet panel	40% (4,373)	39%	39%	42%	39%
Household income per month, column % of non-missing (n)					
€2,000 or less	31% (3,334)	38 %	34 %	29 %	33 %
€2,001-€4,000	47% (5,025)	47 %	48 %	47 %	47 %
€4,001 or more	21% (2,266)	15 %	19 %	24 %	20 %
All non-missing, n	*10,625*				
Missing, n	*176*				
Marital status, column % of non-missing (n)					
Couple (married or co-habiting)	64% (6,932)	64 %	64 %	66 %	60 %
Other (living alone, divorced, separated or widow)	36% (3,841)	36 %	36 %	34 %	40 %
All non-missing, n	*10,773*				
Missing, n	*28*				

[a] Self-reported prescription medication use during preceding year (based on the question on financial difficulties)

[b] Respondent could choose several illnesses.

[c] No missing observations, because the study population excluded responses with missing data on the variables used in the main analyses.
