# Supplementary material for: Citizens’ opinions and experiences related to costs and reimbursements for medications in times of retrenchment: cross-sectional population surveys in 2015 and 2017
Source: Int J Equity Health. 2022 Mar 9;21:33. doi: 10.1186/s12939-022-01631-6 (PMC8905281; doi:10.1186/s12939-022-01631-6)
Supplement: Supplementary file 5 — Additional file 5. Use of and spending on prescription medicines, and opinions on the fairness of the reimbursement system in the study population in total and by exposure group in the pooled 2015 and 2017 data. [file 12939_2022_1631_MOESM5_ESM.pdf]

Additional file 5. Use of and spending on prescription medicines, and opinions on the fairness of the reimbursement system in the study population in total and by exposure group in the pooled 2015 and 2017 data.

		Exposure to reimbursement policies and medicine use (exposure groups)			
	**All**	**Diabetes**	**Eligibility (excl. Diabetes)**	**Others, with prescription medicine use**	**Others, no prescription medicine use**
Total, row % (n)	100% (10,801)	11% (1,206)	23% (2,459)	52% (5,635)	14% (1,501)
Statement: Reimbursements for medicine expenses are fair and just.					
Fully agree	6% (690)	8%	8%	5%	7%
Fairly agree	28% (2,993)	25%	27%	29%	27%
Fairly disagree	26% (2,803)	28%	27%	27%	19%
Fully disagree	16% (1,763)	24%	18%	15%	11%
Don't know/ missing[a]	24% (2,552)	14%	19%	24%	36%
During the last year, have you had financial difficulties in buying medications prescribed to you by a doctor?[b]					
I haven't used prescription medications	14% (1,533)	1%	1%	0%	100%
I have had no difficulties	72% (7,725)	69%	77%	89%	0%
I have had some difficulties	13% (1,366)	26%	19%	10%	0%
I have had plenty of difficulties	2% (177)	3%	3%	1%	0%
Estimate how much you spend on prescription medications in one year?					
I don't use prescription medications	12% (1,345)	1 %	<1%	3 %	78 %
Less than €100	30% (3,216)	6 %	13 %	45 %	19 %
€100-299	28% (3,021)	25 %	33 %	33 %	1 %
€300-599	19% (2,021)	39 %	33 %	13 %	<1%
€600 or more	7% (727)	19 %	14 %	3 %	<1%
Don't know / missing[a]	4% (471)	9 %	7 %	3 %	1 %
Last year, did your prescription medicine expenses exceed the annual co-payment ceiling?					
No	88% (9,521)	70 %	78 %	94 %	99 %
Yes	6% (643)	15 %	13 %	3 %	<1%
Don't know / missing[a]	6% (637)	15 %	9 %	4 %	1 %

[a] Missing answers and "I don't know"-options not distinguishable in the data.
[b] No missing observations, because the study population excluded responses with missing data on the variables used in the main analyses.
